# Oncoplastic Breast-Conserving Surgery Outcomes in the Hispanic Population at a Large County Hospital

**DOI:** 10.7759/cureus.79932

**Published:** 2025-03-02

**Authors:** Amanda Daoud, Kendall A Vignaroli, Aldin Malkoc, Lana Mamoun, Jaclyn R Cerceo, Kevin Perez, Angel Guan, So Un Kim, Amira Barmanwalla, Judi Anne B Ramiscal

**Affiliations:** 1 General Surgery, Arrowhead Regional Medical Center, Colton, USA; 2 Surgical Oncology, Arrowhead Regional Medical Center, Colton, USA

**Keywords:** ethnicity, malignant neoplasm of breast, mammoplasty, mastopexy, oncoplastic breast conserving surgery, postoperative outcomes, surgical disparities

## Abstract

Objectives: Compared to standard breast-conserving surgery (BCS), oncoplastic breast-conserving surgery (OBCS) allows for improved tumor exposure and higher volume tissue resection. OBCS also has lower rates of tumor recurrence and need for re-resection, superior cosmetic outcomes, improved patient satisfaction, and decreased postoperative breast volume, leading to lower doses of postoperative radiotherapy. Additionally, OBCS has no difference in postoperative complications or overall survival compared to BCS. While widespread use of OBCS has increased, racial disparities continue to exist in the utilization of OBCS, and there is little research investigating how race affects OBCS outcomes. To that end, our study aimed to compare outcomes between Hispanic and non-Hispanic patients undergoing OBCS at a large county hospital.

Methods: A retrospective review was performed of adult patients who underwent OBCS at a county hospital in Southern California. Subjects were divided into Hispanic and non-Hispanic groups, and the primary outcomes included the need for re-excision, hematoma or seroma formation, wound infection, areolar loss, skin necrosis, and delayed wound healing.

Results: The Hispanic group had a lower rate of delayed wound healing (p=0.022). Otherwise, there were no significant differences in demographics or outcomes between the two populations, including the need for re-excision, hematoma or seroma formation, wound infection, areolar loss, and skin necrosis.

Conclusion: OBCS is a valuable option when considering reconstruction for lumpectomy in Hispanic populations. The benefits of OBCS should not be understated and should be considered for all patients who are appropriate candidates.

## Introduction

Breast cancer is the most common cancer both in the United States (US) and among women globally, and its burden is expected to increase over the next 20 years [1,2]. While advancements in screening and treatment modalities have improved survival and survivorship, these improvements have not been appreciated proportionately in all races and ethnicities within the US [1].

Oncoplastic breast-conserving surgery (OBCS), which combines resection of breast cancer with plastic reconstruction, all completed in one procedure, is included amongst the recent innovations in breast cancer treatment. OBCS has many benefits over traditional breast-conserving surgery (BCS). When compared to BCS, OBCS provides improved exposure to tumors [3], which allows for the resection of higher volumes of breast tissue [3-8] without compromising aesthetic outcomes [9]. Subsequently, OBCS has lower rates of positive margins requiring re-resection [4,6,7,9-11] and lower rates of cancer recurrence [5,10]. Furthermore, the decreased postoperative breast volume achieved with OBCS generally requires lower doses of postoperative radiotherapy [4,7]. Despite these reported benefits, OBCS still shows no difference in major postoperative complications [10,12] or overall survival [4,5,12] compared to BCS and boasts superior cosmetic outcomes [3,4,6-9,12,13] and improved patient satisfaction [3,6,7,9,14].

OBCS was first utilized in the 1990s [15], and its use has increased more than three-fold in the last decade [16,17]. Historically, there have been racial disparities in access to surgical innovations [18], and the racial disparity in access to surgical treatment of breast cancer is no different. Racial disparities have been shown to exist both in rates of reconstruction after mastectomy in the US [19-22] as well as in postoperative complications after postmastectomy breast reconstruction [23]. While racial disparities also exist with access to OBCS, this discrepancy has decreased in the last decade, with the reported racial index of disparity improving from 23% to 7.6% between 2008 and 2019 [17]. Despite this decrease, it has yet to be seen if equal care and outcomes are being achieved. Little research has investigated if and how race affects outcomes in OBCS. To this end, this study aimed to compare the outcomes of OBCS between Hispanic and non-Hispanic patients.

## Materials and methods

Study design and participants

A retrospective review was conducted on patients who underwent OBCS for breast cancer between October 2021 and September 2023 at a single institution. The patients included in this study were 18 years or older who were diagnosed with breast cancer as the primary diagnosis through biopsy and identified using the International Classification of Diseases, Ninth and Tenth Revisions (ICD-9, ICD-10) codes [24].

All patients had undergone OBCS for their initial oncologic resection, including mammoplasty or mastopexy. All surgeries were performed by a single complex general surgical oncology trained surgeon at one single county hospital in California, US. Reduction mammoplasty was carried out via a wise incision first in the cancer-containing breast and then in the non-oncologic breast. The skin was de-epithelialized and a lumpectomy was performed; however, this lumpectomy specimen was much larger than standard to account for the breast reduction. Equal weights of breast tissue were resected from similar locations of both breasts in order to create symmetry. Mastopexy was conducted first in the cancer-containing breast and then in the non-oncologic breast through this same wise pattern incision so that the nipple areolar complex would appear lifted on either side. In both mammoplasty and mastopexy procedures, surgical clips were placed at the site of the partial mastectomy in the cancer-containing breast in case of positive margins in need of future re-resection. Axillary lymph node biopsy or axillary lymph node dissection was carried out when indicated in both mammoplasty and mastopexy procedures.

Patients were then divided into Hispanic and non-Hispanic patient groups, as defined by patient-reported information. Among these two groups, patient demographics including age at the time of surgery, body mass index (BMI), tumor type, tumor size, and comorbidities including the presence of hypertension, diabetes, and tobacco use were compared. The primary outcomes studied included the need for re-excision due to positive margins, hematoma formation, seroma formation, wound infection, areolar loss, skin necrosis, delayed wound healing, and wound dehiscence. All primary outcomes were evaluated and determined at follow-up appointments in the first one to four weeks after surgery. The need for re-excision was determined by the presence of a margin of less than 2 mm for ductal carcinoma in situ (DCIS), or a positive margin for invasive tumors, as described on the post-excision pathology report.

Data collection 

Ethical approval and approval for data gathering and analysis was obtained from the Arrowhead Regional Medical Center Institutional Review Board (approval number #23-57). After obtaining approval, the electronic medical record was searched for all adult patients who underwent mammoplasty or mastopexy for breast cancer between October 2021 and September 2023, which included 41 patients in total. Demographics in this study were extracted from the data reported in each patient’s pre-operative evaluation. Tumor size was determined as reported on pre-operative ultrasound, and tumor type was determined as reported on pre-operative biopsy. All data was collected and stored in an encrypted and password protected Microsoft Excel (Microsoft Corp., Redmond, US) document.

Statistical analysis

Data was analyzed using the IBM SPSS Statistics version 27.0 (IBM Corp., Armonk, US) software. Continuous data are presented as means and standard deviations and categorical data are presented with frequencies and proportions. Univariate analyses were performed using chi-squared tests for the categorical data. Continuous data was analyzed using Mann-Whitney U tests. All statistical tests were performed as two-sided. Statistical significance was set at p<0.05. 

## Results

Of the 41 patients evaluated, all 41 patients (100%) were female. In total, 30 patients (73%) identified as Hispanic, while 11 patients (27%) did not self-identify as Hispanic and were therefore considered non-Hispanic. In the non-Hispanic group, two patients (5%) identified as Black or African American, two patients (5%) identified as Asian, one patient (2%) identified as American Indian or Alaska Native, and six patients (15%) identified as White.

In the Hispanic group, 24 patients (80%) were diagnosed with invasive ductal carcinoma, and in the non-Hispanic group, nine patients (82%) were diagnosed with invasive ductal carcinoma (p=0.896). Notably, the most common tumor marker expression was estrogen receptor positive (ER+), progesterone receptor positive (PR+), and human epidermal growth factor -2 negative (HER2-), with 14 patients (47%) in the Hispanic group and seven patients (64%) in the non-Hispanic group (p=0.933). The average tumor size in the Hispanic group was 19.6 mm ± 11.1 mm, and the average tumor size in the non-Hispanic group was 28.8 mm ± 26.4 mm (p=0.148). There was no significant difference in the history of smoking, hypertension, and diabetes mellitus (p=0.361, p=0.126, p=0.909) between the two groups. The average age in the Hispanic group was 55.8 years ± 9.1 years and the average age in the non-Hispanic group was 58.4 years ± 8.6 years (p=0.416). The average BMI was 29.86 kg/m^2^ ± 5.3 in the Hispanic group and 30.98 kg/m^2^ ± 4.91 in the non-Hispanic group, respectively (p=0.549) (Table 1).

**Table 1:** Patient characteristics ^a ^Analyzed with independent-groups t test. All other comparisons with chi-square test. ^b ^No statistics are computed because the variable is a constant. BMI: Body mass index

Patient characteristics	Hispanic patients (N=30)	Non-Hispanic patients (N=11)	p-value	
Female	30 (100%)	11 (100%)		.^b^
Age, years, mean^a^	55.8 ± 9.1	58.4 ± 8.6		0.416
BMI, kg/m^2^, mean^a^	29.86 ± 5.3	30.98 ± 4.9		0.549
Hypertension	13 (43%)	6 (55%)		0.361
Diabetes	5 (17%)	4 (36%)		0.126
History of smoking	5 (17%)	2 (18%)		0.909
Tumor size, mm, mean^a^	19.6 ± 11.1	28.8 ± 26.4		0.148
Invasive ductal carcinoma	24 (80%)	9 (82%)		0.896

There was no significant difference in the need for margin re-excision in these groups: eight patients (27%) in the Hispanic group and three patients (27%) in the non-Hispanic group (p=0.969). There were no significant differences in postoperative hematoma formation (three patients (10%) in the Hispanic group vs. two patients (18%) in the non-Hispanic group, p=0.478) or postoperative seroma formation (three patients (10%) in the Hispanic group vs. one patient (9%) in the non-Hispanic group, p=0.931). There were zero instances of postoperative infection, skin necrosis, or areolar loss documented in either group. The Hispanic group had a significantly lower rate of delayed wound healing: one patient (3%) in the Hispanic group compared to three patients (27%) in the non-Hispanic group (p=0.022) (Table 2).

**Table 2:** Postoperative outcomes ^b ^No statistics are computed because the variable is a constant.

Postoperative outcomes	Hispanic patients (N=30)	Non-Hispanic patients (N=11)	p-value	
Margin re-excision	8 (27%)	3 (27%)		0.969
Hematoma formation	3 (10%)	2 (18%)		0.478
Seroma formation	3 (10%)	1 (9%)		0.931
Wound infection	0	0		.^b^
Areolar loss	0	0		.^b^
Skin necrosis	0	0		.^b^
Delayed wound healing	1 (3%)	3 (27%)		0.022

## Discussion

Apart from a lower rate of delayed wound healing in the Hispanic group, we found no statistically significant difference in postoperative outcomes between Hispanic and non-Hispanic patients after undergoing OBCS. A comparable study evaluated outcomes in OBCS in African women and found that seroma was the most common postoperative outcome and invasive ductal carcinoma was the most common histology, similar to our findings [25]. Additionally, Foley et al. compared OBCS to mastectomy with reconstruction and found that their majority non-Caucasian cohort (African American and Hispanic women) had no statistically significant differences in patient reported outcomes between the two operative approaches [26]. These studies serve to reinforce our findings that despite issues with access, underserved minority populations (Hispanic patients in our case) have equivalent outcomes to the general population. 

The social and psychological benefits of OBCS cannot be understated. Patients undergoing OBCS have been found to have improved quality of life compared to those undergoing mastectomy [27]. In fact, oncoplastic surgery has shown the most favorable outcomes when compared to other reconstructive procedures when evaluating patient reported outcome measures [28]. Furthermore, OBCS can even be utilized in patients who have undergone prior breast reduction [29]. This information can be used to advocate for the education and advancement of OBCS at community, county, and academic hospitals so that OBCS can be considered for all patients who are appropriate candidates.

This study was limited by its small sample size, and a future study with a larger sample size is needed to corroborate our findings. Furthermore, this study was conducted at a county hospital that treats a primarily Hispanic population and thus has greater access to resources to support Hispanic patients, including access to necessary translators and culturally competent care. At the national level, breast cancer is the most common cancer among Hispanic women in the US and the top cause of death for this subgroup [30]. Some of the typical barriers that contribute to these outcomes are low levels of mammogram screening adherence, poor insurance coverage, language barriers, transportation, and work and childcare coverage issues [30]. It would be helpful to repeat our study at a facility that treats a smaller proportion of Hispanic patients, which may provide valuable insights into outcomes based on hospital resources. Additionally, further studies involving a larger, more diverse cohort stratifying these outcomes according to the genetic subtype or receptor status of breast cancer may show unique outcomes and might elucidate whether a certain tumor biology or subtype is more common in Hispanic populations. Some studies have shown that histology, the presence of DCIS, and multi-centric features are more predictive of OBCS failure and need for conversion to mastectomy [31]. These outcomes in the setting of the racial disparities described above will need to be further elucidated.

## Conclusions

With demographics and comorbidities being equal in the two populations of interest, there was no statistically significant difference in our primary postoperative outcomes between Hispanic and non-Hispanic patients who underwent OBCS, with the exception of a lower rate of delayed wound healing in the Hispanic group. While OBCS is typically less readily accessible for underserved Hispanic populations, this demographic has outcomes equivalent to those of non-Hispanic populations. The outcomes of this study support OBCS as a valuable option when considering reconstruction methods for lumpectomy in the Hispanic population, and whenever appropriate, these patients should be offered oncoplastic reconstruction.
